# FLIPPER, a combinatorial probe for correlated live imaging and electron microscopy, allows identification and quantitative analysis of various cells and organelles

**DOI:** 10.1007/s00441-015-2142-7

**Published:** 2015-03-19

**Authors:** Jeroen Kuipers, Tjakko J. van Ham, Ruby D. Kalicharan, Anneke Veenstra-Algra, Klaas A. Sjollema, Freark Dijk, Ulrike Schnell, Ben N. G. Giepmans

**Affiliations:** 1 Department of Cell Biology, University Medical Center Groningen, University of Groningen, A. Deusinglaan 1, 9713 AV Groningen, The Netherlands; 2 Present Address: Department of Clinical Genetics, Erasmus Medical Center, Rotterdam, The Netherlands; 3 Present Address: Department of Internal Medicine (Nephrology), University of Texas Southwestern Medical Center, Dallas, Tex. USA

**Keywords:** Light microscopy, Electron microscopy, Correlated microscopy, Probes, FLIPPER, Horseradish peroxidase

## Abstract

Ultrastructural examination of cells and tissues by electron microscopy (EM) yields detailed information on subcellular structures. However, EM is typically restricted to small fields of view at high magnification; this makes quantifying events in multiple large-area sample sections extremely difficult. Even when combining light microscopy (LM) with EM (correlated LM and EM: CLEM) to find areas of interest, the labeling of molecules is still a challenge. We present a new genetically encoded probe for CLEM, named “FLIPPER”, which facilitates quantitative analysis of ultrastructural features in cells. FLIPPER consists of a fluorescent protein (cyan, green, orange, or red) for LM visualization, fused to a peroxidase allowing visualization of targets at the EM level. The use of FLIPPER is straightforward and because the module is completely genetically encoded, cells can be optimally prepared for EM examination. We use FLIPPER to quantify cellular morphology at the EM level in cells expressing a normal and disease-causing point-mutant cell-surface protein called EpCAM (epithelial cell adhesion molecule). The mutant protein is retained in the endoplasmic reticulum (ER) and could therefore alter ER function and morphology. To reveal possible ER alterations, cells were co-transfected with color-coded full-length or mutant EpCAM and a FLIPPER targeted to the ER. CLEM examination of the mixed cell population allowed color-based cell identification, followed by an unbiased quantitative analysis of the ER ultrastructure by EM. Thus, FLIPPER combines bright fluorescent proteins optimized for live imaging with high sensitivity for EM labeling, thereby representing a promising tool for CLEM.

**Electronic supplementary material:**

The online version of this article (doi:10.1007/s00441-015-2142-7) contains supplementary material, which is available to authorized users.

## Introduction

The study of the dynamics of molecules in living cells and the determination of their location at the ultrastructural level are important for understanding the molecular mechanisms underlying cell behavior. Genetically encoded fluorescent proteins (FPs) allow the imaging of molecules and organelles in living cells (Shaner et al. 2007) and fluorescent-based microscopic techniques have been developed that permit near-molecular-resolution imaging of biomolecules (Schermelleh et al. 2010). However, with these so-called super-resolution techniques, generally only one or two distinct molecules are identified and visualized. Electron microscopy (EM), on the other hand, reveals the ultrastructural content of cells down to the molecular level. Unfortunately, protein identification with EM in cellular systems can only be performed in fixed dead cells and techniques used for protein determination usually depend on destructive procedures. Correlated light microscopy (LM) and EM, termed “CLEM”, combines the benefits of these two microscopic techniques, resulting in additional synergistic insight (Brown and Verkade 2010; Giepmans 2008). To identify molecules by CLEM, dedicated probes have been designed that not only give spatiotemporal information in living cells but that can also be detected in their well-preserved ultrastructural surroundings by EM. Ideally, these probes should combine the benefits of (1) the ability to use fluorescence in live cells and (2) their visualization by EM without destructive treatment of cellular membranes.

CLEM is often just an overlay of images sequentially obtained by LM and EM showing ultrastructure. When fluorescence is still needed in EM, a compromise is typically made between fluorescent retention and ultrastructural preservation. Examples are the overlaying of green fluorescent protein (GFP) signals in samples for integrated microscopes (Peddie et al. 2014) or the need for ultrastructural context in so-called super-resolution fluorescence microscopy techniques, based both on the precise localization of proteins, such as for PALM (photoactivated localization microscopy; Betzig et al. 2006) and on laser-induced depletion of fluorescence, so-called STED (stimulated emission depletion; Watanabe et al. 2011). CLEM applications of these overlay techniques are still limited and the use of LM-based identification at EM resolution is less accurate than the use of EM-detectable probes.

The use of EM-detectable probes for CLEM was pioneered with Lucifer yellow to trace neurons (Maranto 1982). Following injection and LM examination, Lucifer yellow was photoconverted by intense illumination, resulting in the production of reactive oxygen species (ROS). When this technique is performed in the presence of diaminobenzidine (DAB), DAB polymers will form and precipitate in close (nanometer range) apposition to the fluorophore (Sosinsky et al. 2007). During subsequent EM preparation, which includes osmium fixation, electron-dense osmium will bind to these precipitates, which will then stand out in EM. Affinity-targeted photoconversion was first explored by using fluorescent ceramide trapped in the Golgi (Pagano et al. 1989) and later, proteins were also identified by specifically targeting the eosin fluorophore to the actin cytoskeleton (Deerinck et al. 1994). Immunotargeting for CLEM has not only been performed with small fluorophores but also with fluorescent particles, such as quantum dots. These nanoparticles are extremely bright and their electron-dense core is readily detectable by EM. Both pre-embedding (Giepmans et al. 2005; Nisman et al. 2004; see also Vu et al. 2015) and post-embedding (Izdebska et al. 2013) labeling for CLEM are feasible with quantum-dot-conjugated antibodies. Alternatively, both a fluorophore and gold can be coupled to an antibody. For pre-embedding labeling, nanogold that needs signal amplification for EM detection can be used, whereas for post-embedding labeling, colloidal gold combined with fluorophores might be an option (for reviews, see Sosinsky et al. 2007; Fabig et al. 2012). Several approaches have been developed to quantify particle-based detection in electron micrographs but the field of view is typically very limited to allow detection of the nanoparticles, which are usually immuno-targeted to proteins of interest (for a review, see Mayhew 2015 in this issue). The application of genetically encoded fluorescent tags, of which GFP (Tsien 1998) is the best example, revolutionized the imaging approaches possible. However, GFP is not generally suitable for photoconversion in order to generate DAB precipitates, although successful application has been shown (Grabenbauer et al. 2005; Grabenbauer 2012). Therefore, other genetically encoded techniques have been developed, starting with a tetracysteine motive with high affinity for biarsenical-conjugated dyes. One of these, named ReAsH, has proven to be extremely efficient for photoconversion by direct illumination (Gaietta et al. 2002). The affinity step, however, involves a balance between specific binding efficiency and a non-specific background. This obviously accounts for all affinity-based techniques, including the immunolabeling discussed above. In the case of the tetracysteine/ReAsH, specificity is increased by optimizing the affinity (Martin et al. 2005) and by using a Förster/fluorescence energy resonance transfer (Arai and Nagai 2013) by exciting genetically encoded GFP in tandem with the tetracysteine (Gaietta et al. 2006). In a subsequent search for a genuine genetically encoded CLEM tag, mini-SOG has been developed (Shu et al. 2011). The singlet oxygen quantum yield (SO-QY) of miniSOG was originally claimed to be higher than that of ReAsH (Shu et al. 2011). In recent studies, the SO-quantum yield (QY) has been argued to be lower (Pimenta et al. 2013; Ruiz-Gonzalez et al. 2013) but transformation-inducing irradiation leads to a higher QY (Ruiz-Gonzalez et al. 2013), explaining the applicability of miniSOG for CLEM (Boassa et al. 2013; Shu et al. 2011). In order to boost the fluorescence of the relatively dim mini-SOG, it has been used in tandem with FPs (Shu et al. 2011).

In addition to photoconversion, enzymatic reactions for the generation of DAB polymers have been exploited. For EM examination only, horseradish peroxidase (HRP) has been used to enhance the electron density of cells following micro-injection (Valtschanoff et al. 1992) or expression in the Golgi apparatus (Connolly et al. 1994) and to localize proteins in the endocytic pathway (Luik et al. 2006; Sunio et al. 1999). More recently, a genetically encoded EM reporter allowing identification of features in the cytoplasm has been developed based on ascorbate peroxidase (Martell et al. 2012) and optimized for higher activity (Lam et al. 2015). This homodimer, called APEX, has been chosen because of its independence of disulfide bridge formation and calcium and will therefore, in principle, allow the detection in both reducing and oxidizing compartments. To prevent dimerization, monomeric versions have been created by introducing specific mutations based on the crystal structure, although this has been achieved at the expense of activity. For CLEM examination, either immunofluorescence or a tandem binding between APEX and mCherry or GFP has been used (Martell et al. 2012).

 We designed a combinatorial probe for CLEM that uses the best of two worlds by combining optimized FPs with highly enzymatically active HRP. With this probe, called FLIPPER (*fl*uorescent *i*ndicator and *p*eroxidase for *p*recipitation with *E*M *r*esolution), several organelle systems can be detected by both LM and EM. With FLIPPER, complete tissue-culture dishes can be straightforwardly labeled for both LM and EM. Here, we combine this strategy with mosaic LM and large-scale EM (Faas et al. 2012; Kuwajima et al. 2013; Ravelli et al. 2013), known as nanotomy, and present quantitative imaging for EM with FLIPPERs.

## Materials and methods

### Molecular cloning

Green fluorescent Golgi-FLIPPER was cloned by replacing the tetracysteine from ManII-GFP-4C (Gaietta et al. 2006) by HRP amplified by the polymerase chain reaction (PCR) from HRP-Stim1 (Luik et al. 2006), a kind gift of R.S. Lewis (Stanford University Medical School), with primers F: GATCGGATCCATGCAGTTAACCCCTACA, and R: GATCGCGGCCGCCTCGAGTTATCCTCCTCCCCTAGAGTTG by using the *Bam*HI and *Not*I sites. With F: AGTCGAATTCATGGTGAGCAAGGGC and R: AGTCGGATCCCTTGTACAGCTCGTC, mOrange2 (Shaner et al. 2008), mCherry (Shaner et al. 2004; both kindly provided by R.Y. Tsien, University of San Diego, California), or mTurquoise2 (a kind gift of T.W.J. Gadella, University of Amsterdam; Goedhart et al. 2012) were amplified and inserted into the construct above replacing enhanced GFP (EGFP) by using *Eco*RI and *Bam*HI. For the endoplasmic reticulum (ER)-FLIPPERs, pDsRed2-ER (632409; clontech) was used. First, the DsRed2-ER fragment was ligated into the *Nhe*I/*Xho*I sites of pCDNA3-ManII-GFP-4C plasmid (Gaietta et al. 2006). Next, various colors of FLIPPERs were ligated in frame with the calreticulin fragment at the *Age*I site by using primers F: GATCACCGGTCGATGGTGAGCAAGGGCGA and R: GATCCTCGAGTTACAGCTCGTCCTTTCCTCCTCCCCTAGAGTTG, ensuring a C-terminal KDEL sequence. The *Eco*RI—*Not*I fragment of Golgi-FLIPPER was ligated in frame with the *Eco*RI site of EpCAM (epithelial cell adhesion molecule) at amino acid 263 by using F: TAATACGACTCACTATAGGGA and R: GATCCTCGAGTTAACCCTGCATTGAGAATTC on EpCAM, resulting in EpEx-FLIPPER. All EpCAM plasmids used have been described previously (Schnell et al. 2013).

### Cell culture, transfection and confocal microscopy

Cells were maintained in Dulbecco’s modified Eagle’s medium supplemented with 5 % fetal calf serum (FCS) and penicillin/streptomycin and cultured at 37 °C in the presence of 5 % CO_2_. HEK293T cells were transfected with Fugene-6 (Promega) according to the manufacturer’s protocol. For multicolor FLIPPER experiments, cells were cotransfected at day 0 with either FLIPPER-mOrange2 together with EpCAM-GFP or FLIPPER-mOrange2 with mutant EpCAM (C66Y) labeled with mCherry (EpCAM[C66Y]-mCherry). At day 1, cells were trypsinized, mixed in a 1:1 ratio and reseeded in glass-bottom Petri dishes (MatTek). On day 2, cells were imaged live under physiological conditions with a confocal system (Zeiss LSM 780). Brightness and contrast were adjusted for presentation purposes. For spectral analysis, fluorescence spectra were recorded in HeLa cells transfected with FLIPPER plasmids by using PPEI. Fluorescence intensities at various wavelengths were measured by using the gamma mode on a confocal scanning system (Zeiss LSM 780, Plan-Neofluar 63×/1.3 Imm Korr DIC M27 lens). Emmision spectra were recorded in lambda mode creating intensity profiles between 416 and 687 nm in approximately 9 nm intervals. Filters and beam splitters used for mTurquoise2, GFP, mOrange2 and mCherry were MBS 458, MBS 458/514, MBS 488 and MBS 488/561, respectively, with a 412–691 filter. Mean image intensities were measured in the imaging data by using Image J Fiji (plot z-axis profile) and normalized to the highest fluorescence intensity measured for the respective fluorescent protein (100 %), yielding relative fluorescence intensity (RFI) plotted as in the graph shown.

By using an automated stage, large high-magnification fields of view were generated by automatically stitching (mosaic) 8 × 8 images recorded in tile scan mode (LCI Plan-Neofluar 63×/1.3 Imm Korr DIC M27). GFP, mOrange2 and mCherry were imaged by using 488-, 514- and 594-nm lasers, respectively, while recording non-overlapping emission. After removal of the culture medium, the cells were fixed with 4 % paraformaldehyde/0.1 % glutaraldehyde in 0.1 M cacodylate buffer for 20 min at room temperature, washed with 0.1 M cacodylate buffer and imaged again in this buffer. With the 20× objective, 5 × 5 tiles were imaged at 1024 × 1024 pixel resolution resulting in an area of approximately 2 × 2 mm.

### DAB polymerization

After the imaging step, cells were washed in phosphate-buffered saline (PBS). HRP visualization by DAB polymerization was performed by using standard protocols. Briefly, 5 mg DAB was dissolved in 10 ml PBS or TRIS-buffered saline; the solution was filtered and, after being mixed with H_2_O_2_ (3 μl 30 %), was added to the cells. Typically after 5–10 min, depending on the visibility of the reaction product, the reaction was stopped by washing away the DAB and the embedding procedure was carried out.

### Electron microscopy

After the DAB reaction, cells were incubated with 1 % OsO_4_ in 0.1 M cacodylate buffer (30 min, 4 °C). After being washed with water, the cells were dehydrated through an increasing graded ethanol series and left overnight at room temperature in a 1:1 mixture of ethanol/Epon, which was replaced by pure Epon (3×) and, in the final step, polymerized at 58 °C. The cover glass of the imaging dish was removed with either liquid nitrogen or by using hydrogen fluoride. The imaged area was selected under a stereo microscope by using intrinsic marks. The relevant area containing the cells of interest on the Epon block was sawn out. Either ultrathin (60 nm) sections were obtained and collected on copper grids for classical transmission EM (TEM), or semithin (0.5 μm) sections were cut and collected on a silicon wafer for large-area EM by using the backscatter detector (BSD). In some cases, the sections were contrasted with uranyl acetate and Reynolds lead citrate following standard procedures. TEM images were recorded by using a FEI CM100 at 80 KV with a Morada camera (Olympus-SIS). Unequal illumination of original EM images was corrected in Adobe Photoshop by applying a gradient levels adjustment layer. BSD images were recorded by using a Zeiss Supra55 Scanning Microscope at 3 KV. Large-area scans were generated by using the external scan generator ATLAS (Fibics Canada) and multiple tiles were stitched by using a VE-viewer (Fibics) and exported either as a high-resolution html file or a single TIF.

### Correlated microscopy

The region imaged by LM before processing for EM was identified as previously described (Sjollema et al. 2012). The imaged area was marked under a stereo microscope by making scratches in the Epon with a scalpel. In Adobe Photoshop, the LM image was copied as a layer into the EM image and made 50 % transparent. Transformation of the LM image is necessary to match it to the larger scale of the EM image. Alignment was carried out with the aid of the intrinsic marks in combination with the shape of the cells in both the differential interference contrast (DIC) image and the EM image. Subsequently, differently color-coded cells, representing different experimental conditions in the same experiment, could be correlated to their respective EM ultrastructure. For ER measurements, selected ultrastructural images of cells with either full-length EpCAM (FL) or EpCAM-C66Y were analyzed by using ImageJ Fiji software. In 10 different cells expressing either FL or mutant EpCAM, ER thickness was measured at 10 different positions per cell.

## Results and discussion

**Fig. 1:**
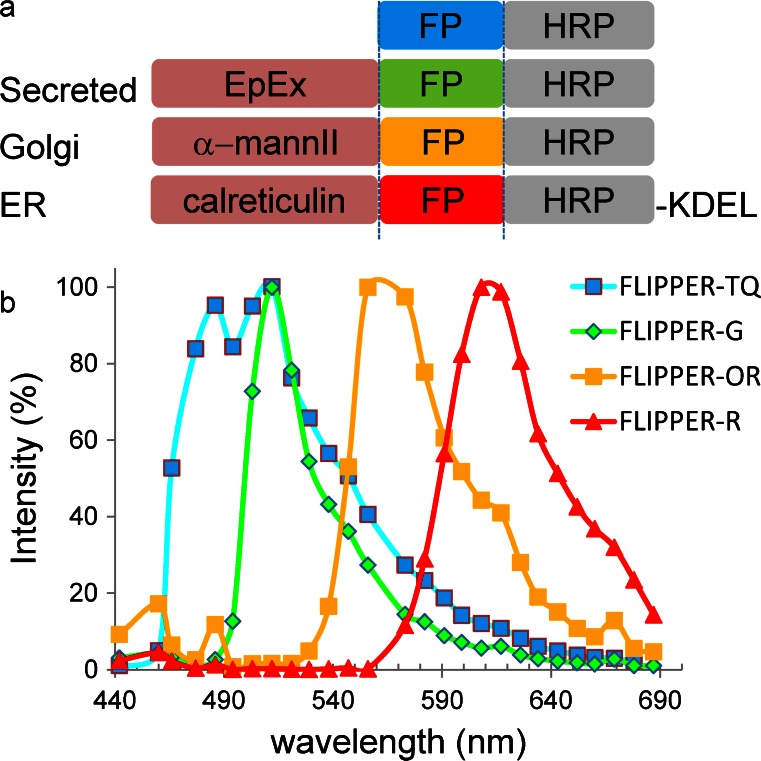
FLIPPER (*fl*uorescent *i*ndicator and *p*eroxidase for *p*recipitation with *E*M *r*esolution), a combinatorial probe for correlated microscopy, combines the advantages of genetically encoded fluorescent proteins (*FP*) and horseradish peroxidase (*HRP*) for correlated light microscopy and electron microscopy (CLEM) with high-quality ultrastructural preservation. **a** Representation of modules emphasizing the straightforward exchange of spectrally different, genetically encoded EM markers (*mannII* mannosidase II, *ER* endoplasmic reticulum). **b** Spectra of FLIPPERs as recorded in transfected cells. The four fluorescent proteins mTurquoise2 (*TQ*, Golgi), enhanced green fluorescence protein (EGFP; *G*, ER), mOrange2 (*OR*, Golgi) and mCherry (*R*, ER), were excited by using 458-, 488-, 514- and 561-nm lasers, respectively

FLIPPER is based on a genetically encoded tag consisting of optimized fluorescent proteins (highly suitable for live-cell imaging) and enzymes to visualize target molecules by EM at high resolution with high quality preservation of the ultrastructure (Fig. 1a). The crystal structure of HRP isozyme C (Gajhede et al. 1997) has revealed that both the N-terminus and C-terminus are distant from the active site, a finding that might be beneficial for making fusions. We made combinatorial fusions (FLIPPER; Fig. 1) to target modules for specific subcellular localization. To target the entire secretory route, we used the extracellular secreted domain of the cell-surface protein EpCAM, named EpEx (Maetzel et al. 2009). In addition, FLIPPERs were fused to a part of mannosidase II; this ensured Golgi localization, as successfully applied previously with the tetracysteine system (Gaietta et al. 2006) and well-proven targeting for the ER based on calreticulin and an N-terminal KDEL sequence (Fig. 1a). The spectral properties of these FLIPPERs as measured inside cells by using a multi-channel lambda scan allowed separation by using distinct excitation and spectral emission properties (Fig. 1b).

**Fig. 2:**
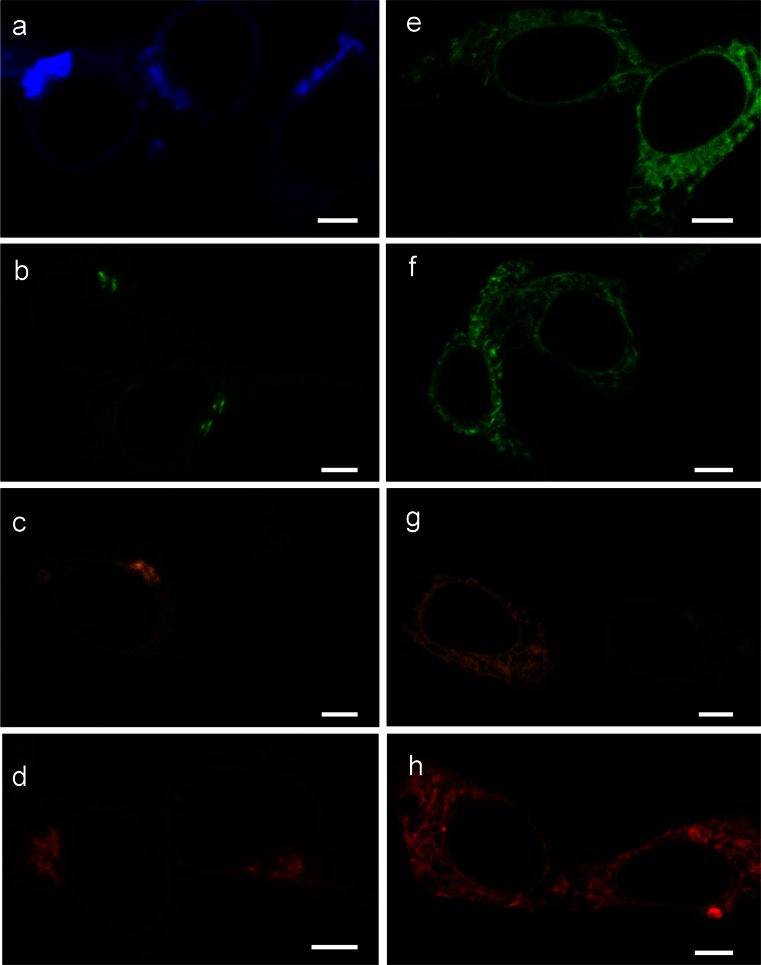
FLIPPER detection by fluorescence microscopy in living cells. Images from living cells taken with a confocal laser scanning microscope. **a–d** Golgi-FLIPPER based on Turquoise2 (**a**), EGFP (**b**), Orange2 (**c**) and mCherry (**d**). **e–h** Secretory FLIPPER based on EGFP (**e**) and ER-FLIPPERs in the same color-code as in **a–d** (**f–h**). Note the typical staining of the Golgi apparatus and ER. *Bars* 5 μm

The various FLIPPERs were expressed in cells and localization was determined in living cells by using confocal microscopy (Fig. 2) one day after transfection. Note that all proteins are expressed from pCDNA3 and will be synthesized in the ER. Therefore, the Golgi-based probes will also pass through this organelle (discussed below). The Golgi-FLIPPERs carrying optimized fluorescent proteins (mTurquoise2, EGFP, mOrange2, and mCherry in Fig. 2a–d, respectively) all visualize the Golgi apparatus with their characteristic distinct wavelengths. Notably, EpEx-FLIPPER is present throughout the secretory route (Fig. 2e) and can even be detected in vesicles in the cytoplasm based on HRP activity (data not shown). The ER-FLIPPERs show characteristic staining of the ER (Fig. 2f–h).

**Fig. 3:**
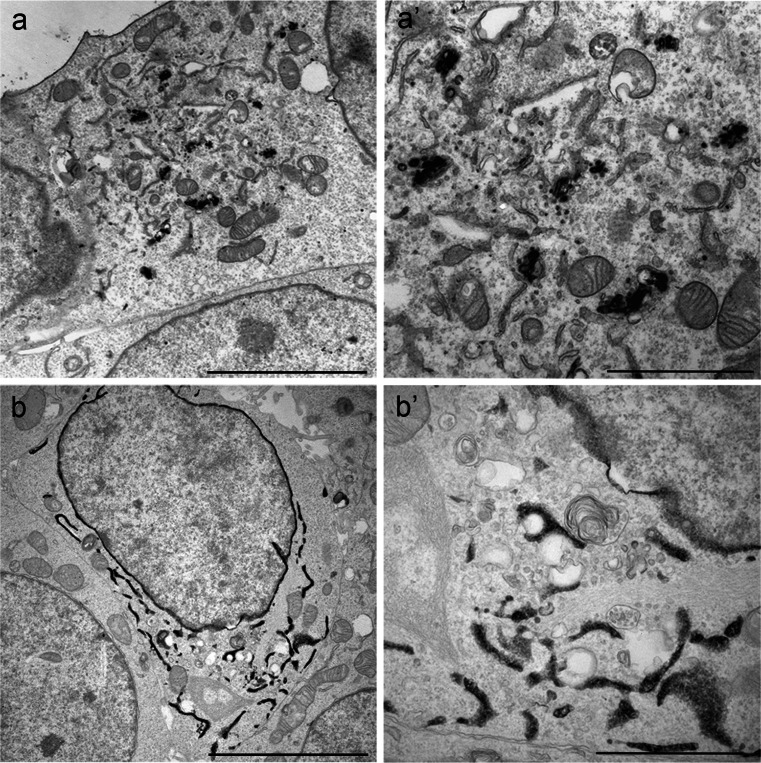
FLIPPER detection using electron microscopy. The *black* DAB deposit created by FLIPPER is readily visible in transfected cells but is absent in non-transfected cells. **a**, **a’** Golgi-FLIPPER. Note that not all Golgi stacks are labeled; this can be explained by the localization of mannosidase II to the medial Golgi stacks but not to the *cis* and *trans* Golgi (Igdoura et al. 1999). **b**, **b’** ER-FLIPPER. Note the absence of precipitate at nuclear pores and the good preservation of ultrastructure. Membranes are readily visible and mitochondrial cristae are crisp. *Bars* 5 μm (**a**, **b**), 2 μm (**a’**, **b’**)

Typically, LM examination and possible selection of expression levels precedes EM analysis. With FLIPPERs, this selection is straightforward. Following a sneak preview at a fluorescence microscope to examine expression in living cells, samples are fixed with a mixture of glutaraldehyde and paraformaldehyde ensuring good fixation. Without the need for permeabilization, DAB polymerization is subsequently performed on the entire sample, as opposed to most photo-conversion protocols. This process can be as short as 15 min and is followed by the traditional EM-preparation steps (osmification, dehydration, embedding and sectioning). The three FLIPPERs with distinct localization were analyzed, revealing the black precipitate in targeted Golgi (Fig. 3a) and ER (Fig. 3b). The ER as marked by the FLIPPER probe is no longer recognizable as the classical membrane-surrounded structure. Because the DAB product fills the complete ER, this masks the membranes and also the ribosomes. When Golgi-targeted FLIPPER is expressed at high levels, it is also detected in the ER, because it will be synthesized en route to the Golgi and the reaction is highly efficient. By using sufficiently low expression levels, however, specific targeting to the Golgi can be achieved. The contrast between transfected cells and non-transfected neighboring cells is easily recognizable (see also Fig. S2 for more examples). Thus, FLIPPERs are easy to use, allow multi-spectral labeling and EM imaging of large areas of interest, potentially aiding in quantitative imaging for CLEM.

We used FLIPPERs to address whether mutations in EpCAM lead to ER dilation. Mutations in the EpCAM gene have been identified as the cause for congenital tufting enteropathy (CTE), a disease presenting with lethal diarrhea attributable to abnormalities in the intestinal epithelium in affected newborns. Previously, we found that all EpCAM mutations in CTE patients led to either secretion of the protein or to retention and accumulation in the ER (Schnell et al. 2013). We hypothesized that ER retention of EpCAM caused ER stress. In some instances, ER stress might result in the widening of the ER lumen (Ravelli et al. 2013).

**Fig. 4:**
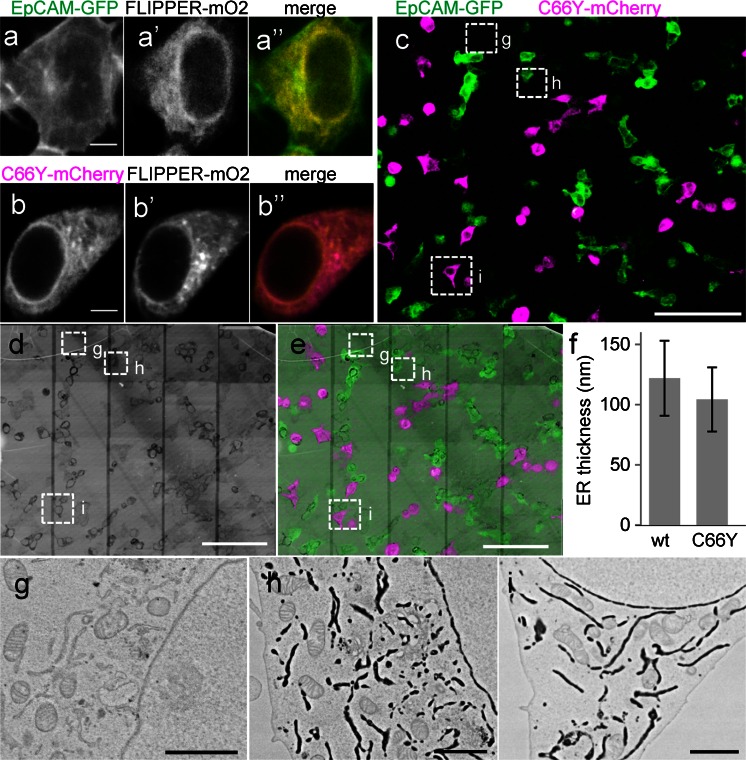
Mix and match. FLIPPER in various experimental conditions within a single dish and quantitative EM based on LM. **a**, **b** Representative cells expressing (**a-a’’**) FL (full-length) EpCAM-GFP (epithelial cell adhesion molecule fused to green fluorescent protein) or (**b–b’’**) mutant EpCAM(C66Y)-mCherry, together with FLIPPER-mOrange2, showing plasma membrane localization of FL EpCAM and ER localization of mutant EpCAM. **c–e** 293T cells transfected with FL EpCAM-GFP and with EpCAM (C66Y) fused to mCherry and visualized as indicated. **f** *Bar graph* indicating thickness of ER in transfected cells as measured by using ER-FLIPPER. *n* = 100 measurements in 10 cells; *error bars* indicate standard deviation. Note that the measurements were made under identical conditions in the same experiment. **g–i** Parts of the *boxed* cells in **c–e**. *Bars* 5 μm (**a**, **b**), 100 μm (**c–e**), 2 μm (**g–i**)

To address quantitatively whether the ER lumen was dilated in cells expressing ER-retained EpCAM mutants, we combined the staining of the ER with FLIPPER-mOrange2 with the spectral identification of cells expressing either FL EpCAM-GFP or point-mutated EpCAM(C66Y)-mCherry (Fig. 4). Samples were first analyzed by large-scale confocal microscopy to identify cells expressing FL or mutant EpCAM, followed by large-scale EM preparation (Fig. S1). The overlay contains information on cell identity, whereas FLIPPER allows for straightforward screening of the ER lumen at the ultrastructural level (Fig. 4; see also Fig. S2 showing additional data at higher resolution). Using this method, we determined ER width at 10 different positions in 10 different cells, i.e., 100 data points per cell type, allowing rapid quantification of the EM data based on CLEM and nanotomy. Based on this experiment, we conclude that the retention of mutant EpCAM in the ER has no effect on ER morphology or luminal width (Fig. 4f). The multicolor LM approach, combined with FLIPPER and nanotomy, has thus proved to be an important tool in quantifying protein effects on certain organelles.

### Concluding remarks and future outlook

Several probes have now been developed, validated and implemented allowing protein identification at both LM and EM levels. Genetic encoding ensures the maximal specificity of targeting of the protein of interest by use of chimeras. Once the DNA has been delivered, cells do not have to be perturbed to pass large molecules such as antibodies inside cells and organelles and thus, the ultrastructure is superiorly preserved compared with that following immune affinity-based techniques, a hurdle for the localization of proteins by EM. The majority of genetically encoded probes that can be detected at both LM and EM levels are based on continuously improving fluorescent proteins and/or osmiophilic DAB precipitates (Table 1).

The genetically encoded tetracysteine/biarsenical system is partially affinity-based and therefore gives background. This system requires reduced cysteines, which can be induced by post-fixation in oxidizing compartments (Gaietta et al. 2006). The technique does allow multi-color labeling, which elegantly has been used to perform pulse-chase at the EM level (Gaietta et al. 2002). Although mini-SOG (Shu et al. 2011) does not need an affinity step, its fluorescence is weaker and the protein is larger than the tetracysteine-biarsenical system. Whereas both of these probes need photoconversion, the enzyme-based APEX and FLIPPER allow for a more simplified deposition of DAB polymers. Importantly, strong fixatives are compatible with the retention of enzymatic activity. The size of these enzyme-based probes is significantly larger than that of the photoconversion-based probes; this might pose a problem for some targets. The engineering of APEX such that it can be used (1) as a monomer and (2) at the cytoplasmic site has led to a successful module but the enzyme activity is significant lower than that of HRP (Martell et al. 2012); this has been partially overcome by engineering APEX2 (Lam et al. 2015). FLIPPER is highly active, making it more likely to be detectable at lower expression levels; however, at this stage, it cannot be used in the cytoplasm. A drawback of DAB precipitation is that only one target at a time can be identified at the EM level. Making use of the available fluorescent colors, we identified cells expressing certain mutant proteins in mixed populations. This now allows the transfection of various cells and the color-coding of these cells. Single cells represent individual experimental conditions but can be treated exactly the same for EM preparation and analysis. In combination with large-scale LM and EM examination in which the cells are identified based on color, these cells can be subsequently matched in the EM data, allowing side-by-side analysis. The photoconvertible probes are typically used for small areas, whereas the enzyme-initiated conversion is typically performed in the entire sample. We optimized microscopic acquisition to be able to image multiple or large areas, including mosaic microscopy, at both the LM and EM level.

**Table 1:** Dedicated genetically encoded probes for correlated light microscopy and electron microscopy (CLEM) based on diaminobenzidine (DAB) deposition. Whereas FLIPPER (*fl*uorescent *i*ndicator and *p*eroxidase for *p*recipitation with *E*M *r*esolution) has been specifically developed for multispectral analysis (this report), other fusions with fluorescent proteins (FPs) have been introduced to enable (APEX; Martell et al. 2012; Lam et al. 2015) or increase (miniSOG; Shu et al. 2011) fluorescence or to obtain more specific photoconversion by using fluorescence energy resonance transfer (FRET; ReAsH; Gaietta et al. 2006)

Probe	Mechanism	Specifics
4Cys-ReAsH	Photoconversion	Includes affinity-step in labeling, very small
miniSOG	Photoconversion	Relatively dim, small
APEX, APEX2	Enzymatic	Weaker than horseradish peroxidase (HRP), cytoplasmic activity
FLIPPER	Enzymatic	Secretory route only, bright and active

The key is to create CLEM probes that are suitable for general use and that are, thus, widely applicable, similar to the achievements for FPs, starting with GFP two decades ago. The combinatorial tag allows not only to perform live-cell imaging but also to construct chimeric proteins including initial fast LM validation by using routine methods. The added values of genetically encoded probes for CLEM are (1) highly specific targeting; (2) high-quality preservation of the ultrastructure; (3) cheap amplification and easy sharing within the scientific community; (4) pre-embedding labeling suitable for three-dimensional EM; (5) compatibility with labeling in living cells in vitro and in vivo, even including animals (Shu et al. 2011). Further efforts will be needed to engineer a cytoplasmic FLIPPER-like module or APEX variants with higher activity and, by extension, higher sensitivity. A future alternative to direct tagging of proteins of interest might be the fusion of genetically encoded CLEM probes to genetically encoded single-chain antibodies to targets (Rothbauer et al. 2006). Finally, the parallel development of probes based on various principles will help current developments in simultaneous labeling of multiple structures in EM (“multi-color EM”) and will allow better quantitative analysis of ultrastructural data.

## Electronic supplementary material

Below is the link to the electronic supplementary material.

ESM 1 (PDF 1364 kb)
