# Supplementary material for: FLIPPER, a combinatorial probe for correlated live imaging and electron microscopy, allows identification and quantitative analysis of various cells and organelles
Source: Cell Tissue Res. 2015 Mar 19;360(1):61–70. doi: 10.1007/s00441-015-2142-7 (PMC4379394; doi:10.1007/s00441-015-2142-7)
Supplement: Supplementary file 1 — (PDF 1364 kb) [file 441_2015_2142_MOESM1_ESM.pdf]

FLIPPER, a combinatorial probe for correlated live imaging and electron microscopy, allows identification and quantitative analysis of different cells and organelles

Jeroen Kuipers, Tjakko J. van Ham, Ruby D. Kalicharan, Anneke Veenstra-Algra, Klaas A. Sjollema, Freark Dijk, Ulrike Schnell & Ben N. G. Giepmans

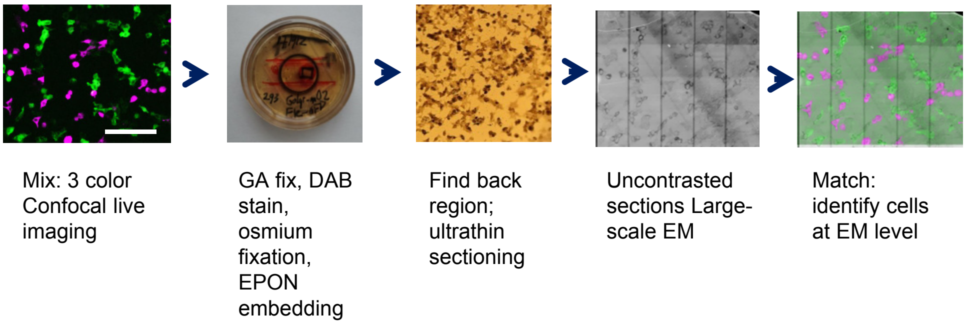

Fig. S1. Mix and match: using FLIPPER for unbiased quantitative EM within a single experiment

Workflow for mix & match as indicated. Different dishes of cells are transfected, trypsinized and mixed to have a heterogeneous cell population. The color of the cell indicates which proteins are expressed. Following fluorescent imaging of living or fixed cells, the sample is prepared for EM. In the epoxy, the region of interested is located and selected. Next, thin sections are made, followed by large scale image acquisition with an EM and finally the LM/ EM images are overlayed. EM analysis of the cells is performed, and color identifies the protein expressed. Bar: 100 µm.

FLIPPER, a combinatorial probe for correlated live imaging and electron microscopy, allows identification and quantitative analysis of different cells and organelles

Jeroen Kuipers, Tjakko J. van Ham, Ruby D. Kalicharan, Anneke Veenstra-Algra, Klaas A. Sjollema, Freark Dijk, Ulrike Schnell & Ben N. G. Giepmans

a. EPCAM(WT); ER-FLIPPER-mOrange2

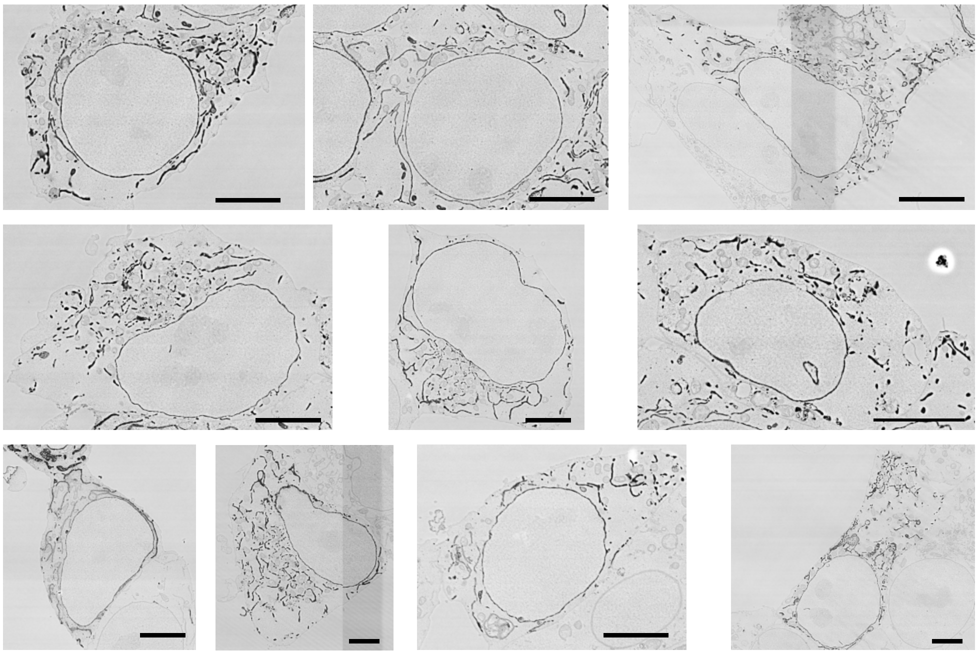

b. EPCAM(C66Y); ER-FLIPPER-mOrange2

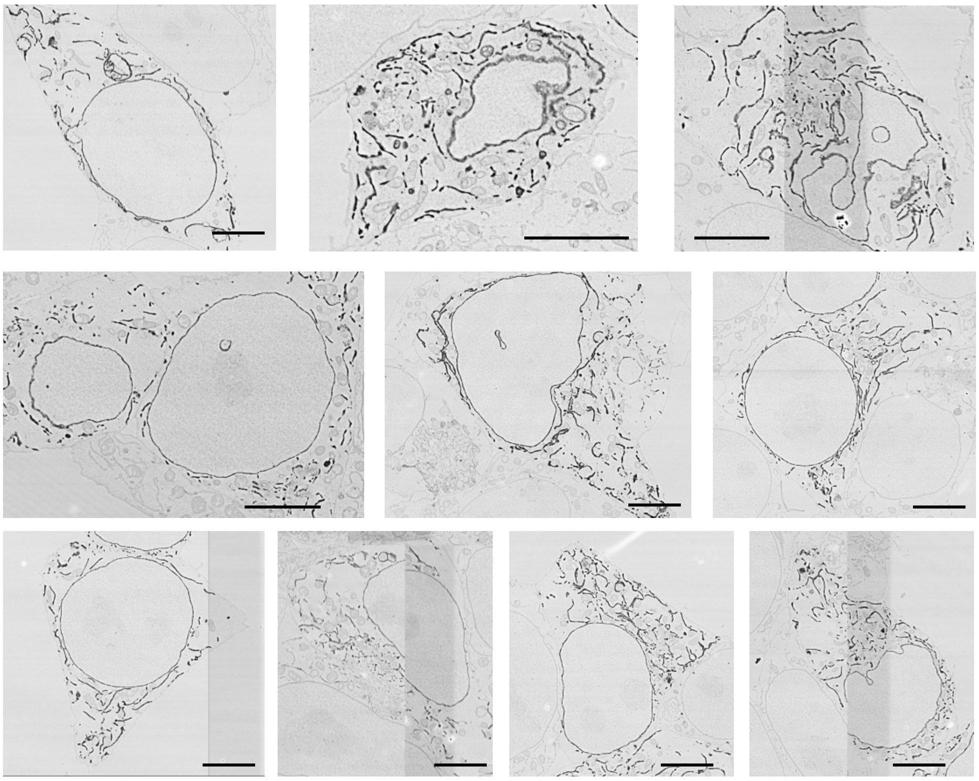

Fig. S2 Mix and match: Cells used for quantification of ER thickness in Fig.4.

Selected cells that were used for quantification as shown in Fig. 4. Bars: 5 μm.
